# Modified Molecular Chain Displacement Analysis Employing Electro-Mechanical Threshold Energy Condition for Direct Current Breakdown of Low-Density Polyethylene

**DOI:** 10.3390/polym13162746

**Published:** 2021-08-16

**Authors:** Minhee Kim, Se-Hee Lee

**Affiliations:** School of Electronic and Electrical Engineering, Kyungpook National University, Daegu 41566, Korea; mylily4530@gmail.com

**Keywords:** bipolar charge transport, breakdown, molecular chain displacement, space charge, threshold energy

## Abstract

In an HVDC environment, space charge accumulated in polymeric insulators causes severe electric field distortion and degradation of breakdown strength. To analyze the breakdown characteristics, here, the space charge distribution was numerically evaluated using the bipolar charge transport (BCT) model, considering the temperature gradient inside the polymeric insulator. In particular, we proposed an electro-mechanical threshold energy condition, resulting in the modified molecular chain displacement model. The temperature gradient accelerates to reduce the breakdown strength with the polarity-reversal voltage, except during the harshest condition, when the temperature of the entire polymeric insulator was 70 °C. The energy imbalance inside the insulator caused by polarity-reversal voltage reduced the breakdown strength by 82%. Finally, this numerical analysis model can be used universally to predict the breakdown strength of polymeric insulators in various environments, and help in evaluating the electrical performance of polymeric insulators.

## 1. Introduction

Increasing demand for eco-friendly energy has led to the development of HVDC cables for low Joule loss in power transmission. Polymeric insulators with high physical and chemical stabilities are widely used in high-voltage power devices [1,2,3]. Polymeric insulators must endure a severe operating environment with a high DC voltage [4,5]. In an HVDC environment, many space charges are accumulated inside the polymeric insulator due to continuously applied one-directional voltage [6,7,8,9]. This space charge causes a distorted electric field, which plays a critical role in lifetime reduction [10,11,12,13]. The study of space charge measurement has been conducted for several decades with various methods, such as pulsed electro-acoustic (PEA), pressure wave propagation (PWP), laser induced pressure pulse (LIPP), and current integration (Q(t)) methods, etc. [14,15,16,17,18,19,20]. Numerical analysis of space charge and electric field has been actively conducted with the conductivity and bipolar charge transport (BCT) models [21,22,23]. With space charge analysis development, studies measuring the breakdown strength and analyzing the mechanisms have been conducted as the critical factor for stability and reliability. The breakdown strengths of polymeric insulators have been studied using numerical and statistical experimental approaches [24,25,26,27]. There are several models to interpret the breakdown mechanisms inside the polymeric insulators, such as the electro-mechanical model, electron avalanche model, molecular chain displacement model, and phase-field model [1,27,28].

In recent studies on numerical breakdown strength prediction employing various models, the space charge effect was not considered in some limits, or only the special cases were dealt with related to the applied voltage and temperature. The breakdown strength of polymeric insulators was analyzed under certain conditions, increasing voltage with constant ramp rate, with a constant temperature in the previous studies. However, in the actual system, the polymeric insulator should endure the various voltage waveforms such as a constant direct current (DC) voltage or polarity-reversal voltage (PRV) for a long time. Additionally, high DC voltage causes a severe temperature gradient inside to change the space charge distribution. These accumulated space charge behaviors greatly affect the breakdown strength of the systems.

Encouraged by the recent research, here, we analyzed the breakdown phenomena of low-density polyethylene (LDPE) under PRV with a temperature gradient. For this purpose, we adopted the BCT model for analyzing space charge. Additionally, we proposed a new evaluation model for analyzing the breakdown phenomenon, including a novel threshold energy condition for the molecular chain scale. We determined the initiation of breakdown phenomena, employing the electro-mechanical threshold energy condition coupled with the molecular chain displacement (MCD) model.

In this study, we found that breakdown strength decreased under PRV with a temperature gradient. As the temperature gradient inside the polymeric insulator increased, the breakdown strength decreased. Additionally, there were differences in breakdown strength and initiation time depending on which temperature was higher, anode or cathode. Notably, we revealed that the breakdown mechanism could be explained by the imbalance of mechanical and electrical energy through our proposed numerical model. With this new approach, we expect this research to contribute to the development of new composites for improved electrical performance.

In Section 2, the numerical analysis conditions and methods are described. In Section 3, the fully coupled finite element analysis for space charge transport is described, incorporating the BCT and the heat transfer equations. Additionally, we discuss the space charge behavior and the resulting electric field distortion related to the electric breakdown issue. In Section 4, a modified MCD model is proposed to predict the electric breakdown in LDPE under HVDC stress by use of the electro-mechanical threshold energy condition, and the breakdown strengths are evaluated under various conditions. Finally, in Section 5, the breakdown mechanism is explained inside the polymer insulator based on the proposed numerical model.

## 2. Analysis Setup and Test Conditions

LDPE is one of the most popular polymeric insulators actively used in HVDC cable systems [29,30]. To implement the proposed method, Poisson’s equation and BCT model were used to analyze the space charge behavior and electric field distortion with 200 μm thick LDPE as a one-dimensional analysis model. The electro-mechanical threshold energy condition, coupled with the MCD models was used to calculate the breakdown strength and decide the breakdown initiation.

**Figure 1:**
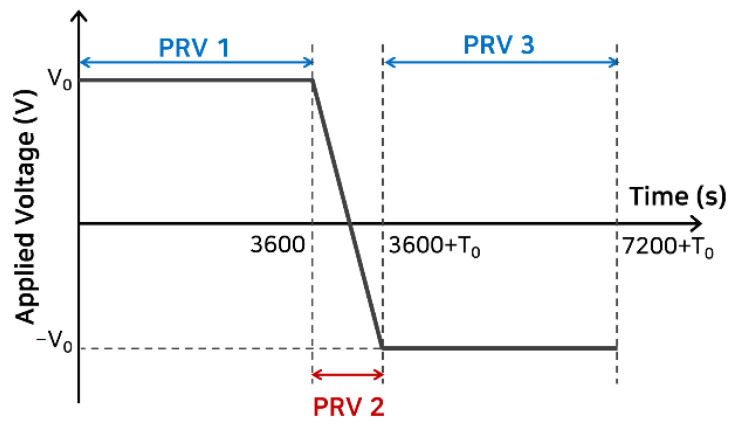
The applied polarity-reversal voltage (PRV) waveform is divided into three parts: PRV 1–3. In PRV 1, constant magnitude voltage (*V*_0_) is applied. In PRV 2, a transition voltage is applied with the same magnitude but in the opposite direction for *T*_0_, 40 s. In PRV 3, the opposite direction voltage is applied for the same time as PRV 1.

When a high DC voltage is applied to the cable, a severe temperature gradient occurs in the polymeric insulator. Here, we analyzed the space charge and electric field distribution over time under various temperatures and applied voltage conditions, as shown in Table 1. As described in Table 1, two types of voltage waveforms were applied to the polymeric insulators: one-directional constant voltage (CV) and polarity-reversal voltage (PRV) with the maximum value, *V*_0_ and *T*_0_ is the voltage polarity transition time, set to 40 s, as depicted in Figure 1 and Figure S1.

The temperature inside the polymeric insulator is also composed of constant (@CT) and temperature gradient (@GT). The temperature of the polymeric insulator was set to 30 °C, 50 °C, and 70 °C, and the temperature gradient was set to 20 °C (30 °C~50 °C and 50 °C~70 °C) and 40 °C (30 °C~70 °C). When there exists a temperature gradient inside, it can be divided into two types: heated anode (@GT-A) and heated cathode (@GT-C).

In this study, we implemented the numerical analysis based on the finite element method. The analysis time interval, ∆*t*, satisfied the Courant–Friedrichs–Lewy condition as ∆*t* < ∆*x*/*μE* for holding numerical stability [22,31,32]. Therefore, the distance through which the charge carrier moved per unit time was smaller than the interval of the finite element mesh.

## 3. Analysis of Space Charge Transport and Electric Field Distribution

### 3.1. Bipolar Charge Transport Model

**Figure 2:**
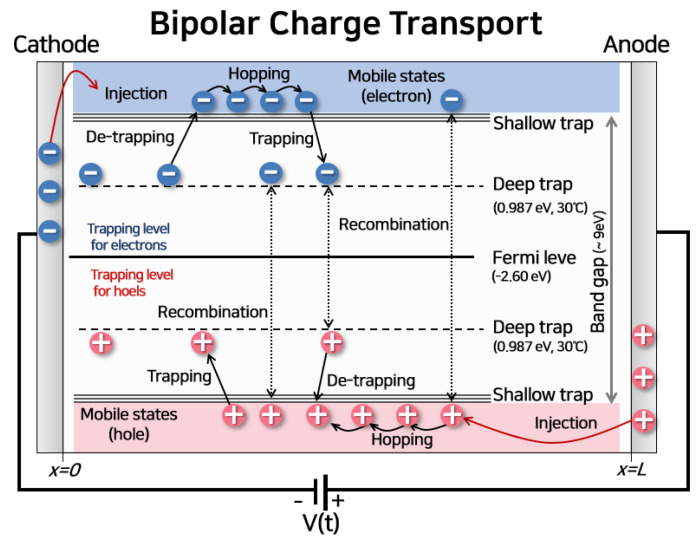
BCT model: The electrons are injected from the cathode, and holes are injected from the anode depending on the electric field strength and temperature. These charges transport according to hopping conduction at the shallow trap, which is the localized energy level. These charges are trapped in the deep trap, and the de-trapping process occurs, contributing to the conduction current. The energy and density of the deep trap change with temperature. In this study, the Fermi level of LDPE was set to −2.60 eV, and the deep trap energy was set to 0.987 eV in 30 °C. The recombinations between trapped electron, free electron, trapped hole, and free hole are also considered.

The BCT model includes the transport process of two types of charge carriers, such as electrons and holes injected from an electrode. The whole transport process of the injected electrons and holes is included in the BCT model, as depicted in Figure 2. Space charges are injected from the electrode to the polymeric insulator following the Schottky thermionic injection mechanism under a high electric field as Equation (1). As described in Equations (2)–(4), the space charge distribution affects the electric field in real-time. Equation (2) is Poisson’s equation, Equation (3) is the continuity equation for space charge and current density, and Equation (4) is the drift equation, including conduction current density and diffusion effects of space charge as [4]:

(1) $$j_{in(e,h)}=AT^{2}\exp(\frac{-\Phi_{A,C}-\sqrt{eE_{A,C}/4\pi \varepsilon_{0}\varepsilon_{r}}}{k_{B}T})$$

(2) $$\frac{\partial^{2}V(x,t)}{\partial x^{2}}=-\frac{\rho (x,t)}{\varepsilon_{0}\varepsilon_{r}}$$

(3) $$\frac{\partial \rho_{e\mu ,h\mu }}{\partial t}+\frac{\partial J_{d}}{\partial x}=S_{e\mu ,h\mu }$$

(4) $$J_{d}(x,t)=\mu_{e,h}(x,t)\rho_{e,h}E(x,t)-D_{f}\frac{\partial \rho_{e\mu ,h\mu }(x,t)}{\partial x}$$

where *J_in_*_(*e*,*h*)_ is the current density by the injected charge carriers at anode and cathode in A/m^2^; *A* is Richardson constant, 1.26 × 10^6^ A/m^2^∙K^2^; *E_A,C_* is the electric field strength at the cathode and anode, respectively in V/m; Φ*_A_*_,*C*_ is the injection barrier height between the electrode and insulator at the cathode and anode, in eV; *k_B_* is Boltzmann constant; *T* is the absolute temperature in *K*; and *e* is the unit charge, 1.6 × 10^−19^ C. The subscripts *e* and *h* expresse the type of the charge carriers, electrons, and holes. *V* is the electric potential in V, *ρ*(*x*,*t*) is the total space charge distribution in C/m^3^, including free mobile electrons, holes, trapped electrons, and trapped holes. *ε*_0_ is the permittivity of vacuum in F/m, and *ε_r_* is the relative permittivity. *ρ_eμ_*_,*hμ*_ is the free mobile charge carrier density in C/m^3^, *J_d_* is the conduction current density in A/m^2^, and *S* is the reaction-generation term for each charge in C/m^3^∙s. The reaction-generation process includes charge recombination, trapping, and de-trapping, as depicted in Figure 2. *μ_e_*_,*h*_ is the mobility of the free mobile charges in m^2^/V∙s, *E*(*x*,*t*) is the electric field in V/m, and *D_f_* is the diffusion coefficient in m^2^/s.

The coefficients used in the BCT model were obtained from previous experimental studies, such as electron and hole mobility, deep trap energy and density, and injection barrier height that varies with temperature [24,25,26,27]. In particular, we assumed that deep trap energy has a single level for simplicity in this numerical analysis model. (The detailed charge transport processes and parameters are included in Supplementary Material, S1).

### 3.2. Heat Transfer Model

The polymeric insulator temperature increases due to the Ohmic heat at the end of the electrode in the HVDC system. Then, a temperature gradient exists across the polymeric insulator. In this numerical simulation, we employed Fourier’s Law to analyze the heat transfer process inside the polymeric insulator as [33]:

(5) $$\rho_{p}C_{p}\frac{\partial T(x,t)}{\partial t}+\frac{\partial q(x,t)}{\partial x}=Q_{0}$$

(6) $$J_{d}(x,t)=\mu_{e,h}(x,t)\rho_{e,h}E(x,t)-D_{f}\frac{\partial \rho_{e\mu ,h\mu }(x,t)}{\partial x}$$

where *ρ_p_* is the mass density of LDPE in 1400 kg/m^3^, *C_p_* is the heat capacity in 2500 J/kg∙K, *Q*_0_ is the heat source in W/m^3^, *k* is the thermal conductivity in W/m∙K, and **q**(*x,t*) is the heat flux density in W/m^2^. *Q*_0_ is caused by the current density and the electric field inside the polymeric insulator. (The detailed parameters are included in Supplementary Material, S1).

### 3.3. Characteristics of Space Charge Transport Resulting in the Maximum Electric Field Strength

**Figure 3:**
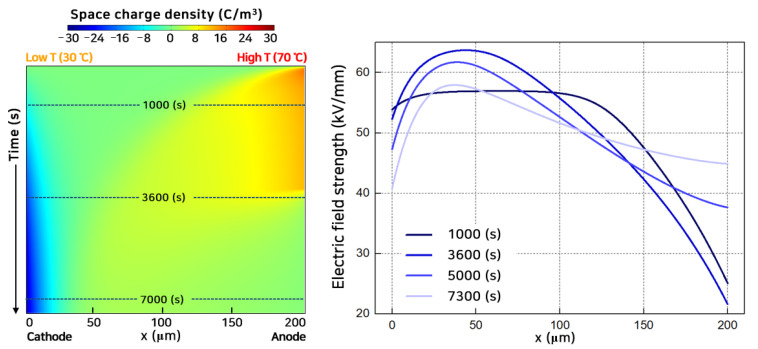
Space charge distribution with time (**left**) and electric field strength (**right**) in CV@GT-A2 and *V*_0_ = 10 kV.

Figure 3 shows the space charge and electric field distribution when the temperature at the anode region was 40 °C higher than that of the other region (CV@GT-A2). Many holes were injected from the anode in the form of packets and penetrated quickly. Electrons injected from the cathode crossed the polymeric insulator with a lower speed than the holes. The electrons were trapped near the cathode with a higher probability than the holes and sequentially lowered the electric field at the cathode. Holes quickly penetrated the polymeric insulator and recombined with the slow-moving electrons actively near the cathode. Therefore, the maximum electric field appeared in the low-temperature region, cathode. (Detailed space charge behavior depicted in Supplementary Material, Figure S4).

**Figure 4:**
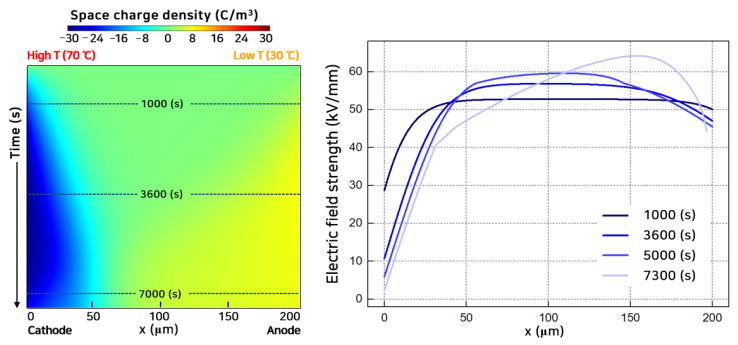
Space charge distribution with time (**left**) and electric field strength (**right**) in CV@GT-C2 and *V*_0_ = 10 kV.

In contrast to Figure 3, Figure 4 shows the space charge and electric field distribution when the temperature at the cathode region was higher than the other region (CV@GT-C2). At the cathode region where the temperature was high, a large number of electrons were injected into the polymeric insulator with high kinetic energy. In the case of an LDPE, the injection barrier height is lower for holes than for electrons [34]. Therefore, holes could inject into the polymeric insulator easier, even at a lower temperature. The electrons move with a higher speed than the holes over the entire polymeric insulator. Compared with Figure 3 (CV@GT-A2), mobile electrons with high kinetic energy could penetrate deeper into the polymeric insulator [35]. Additionally, the zero point of the space charge distribution moved toward the anode. As a result, the maximum electric field was located near the cathode and gradually moved to the anode region. (Detailed space charge behavior depicted in Supplementary Material, Figure S5). As can be seen from Figure 3 and Figure 4, the maximum electric field always appeared at the low-temperature region under CV.

**Figure 5:**
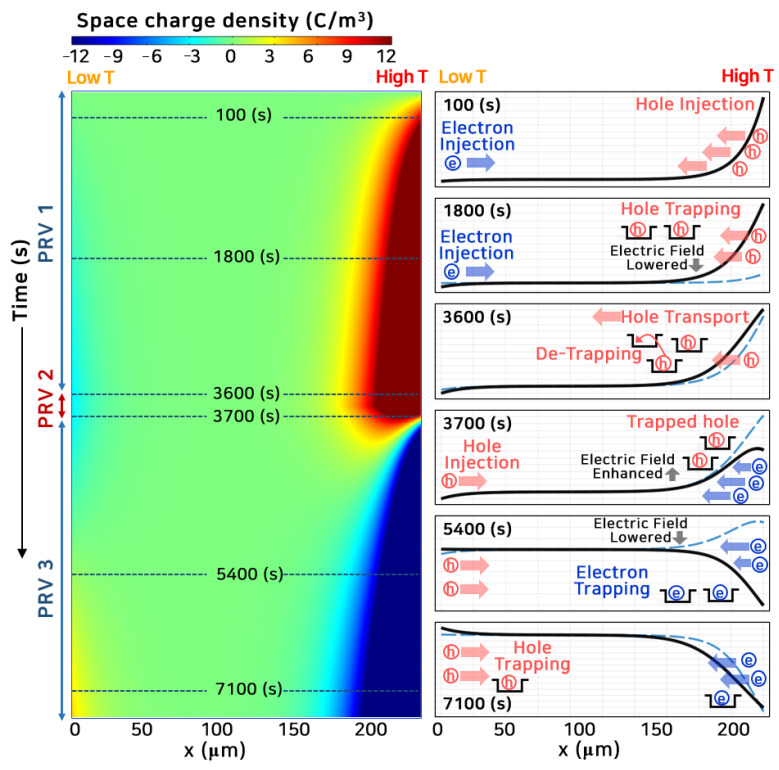
Space charge distribution with time as colored surface (**left**). *x* = 0 sets as a cathode, *x* = 200 μm sets as an anode. The anode and cathode are high- and low-temperature regions, respectively. In PRV 2, the polarity of the voltage is reversed. Total space charge distribution, including free mobile and trapped charge is depicted with time (**right**).

**Figure 6:**
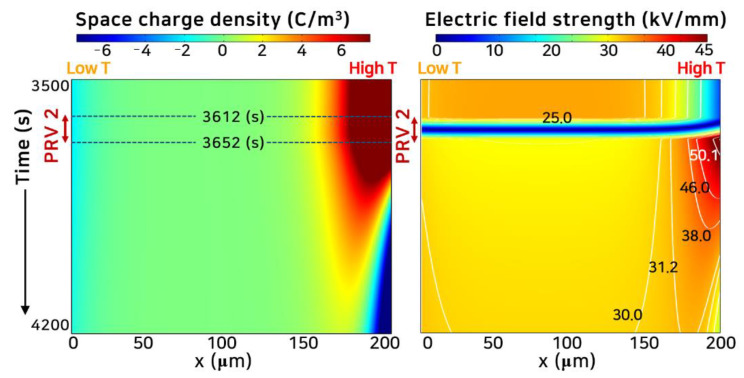
Space charge and electric field distribution with time under PRV. At the beginning of PRV 2, the electric field is close to zero. Even after the voltage polarity is changed, the injection of opposite charges is blocked for a while due to the trapped hetero-charge. Immediately after PRV 2, the electric field strength increases steeply, and then the charge injection with opposite polarity gradually increases. After ~4000 s, the total space charge distribution is completely reversed in its polarity.

Figure 5 and Figure 6 show the space charge behavior and electric field distribution inside the polymeric insulator, where PRV was applied with a temperature gradient. In this case, the temperature gradient was 40 °C (PRV@GT-A2), and *V*_0_ of 6 kV was applied in Figure S1b. First, a positive directional voltage was applied for 3600 s, and voltage polarity was reversed between 3612 and 3652 s in 40 s. Next, the negative directional voltage was applied for 3600 s.

As shown in Figure 5, many holes were injected from the anode, high-temperature region, moving toward the opposite electrode with high kinetic energy. A small number of electrons were injected from the cathode, low-temperature region, compared to the holes. The holes were trapped in a deep trap adjacent to the anode, where many of them were injected. These trapped holes lowered the electric field as the homo-charges, steadily decreasing the number of injected holes. In PRV 1, with a temperature gradient inside the polymeric insulator, the maximum electric field appeared in the low-temperature region under the positive voltage as described in Figure 3 and Figure 4. In PRV 2, the opposite polarity charge was progressively injected from the electrodes, and the space charge distribution was completely reversed around 4000 s. After the voltage polarity was reversed, the maximum electric field strength shifted to the high-temperature region. The space charge distribution in PRV 3 became a mirror image of the space charge distribution in PRV 1, which agreed well with the space charge measurement results [14,36].

Figure 6 shows the detailed space charge and electric field strength in PRV 2. After the polarity of the voltage was reversed in PRV 2, the electric field strength decreased rapidly to zero, and the electric field strength became the minimum during the entire operating time. However, the electric field strength suddenly increased due to the interaction of the hetero-charge accumulated in the vicinity of the electrode and the opposite charge injected after a polarity reversal. This electric field strength had a maximum value of 50.1 kV/mm.

## 4. Prediction of Breakdown Strength with Electro-Mechanical Threshold Energy Condition

### 4.1. Modified Molecular Chain Displacement Model

**Figure 7:**
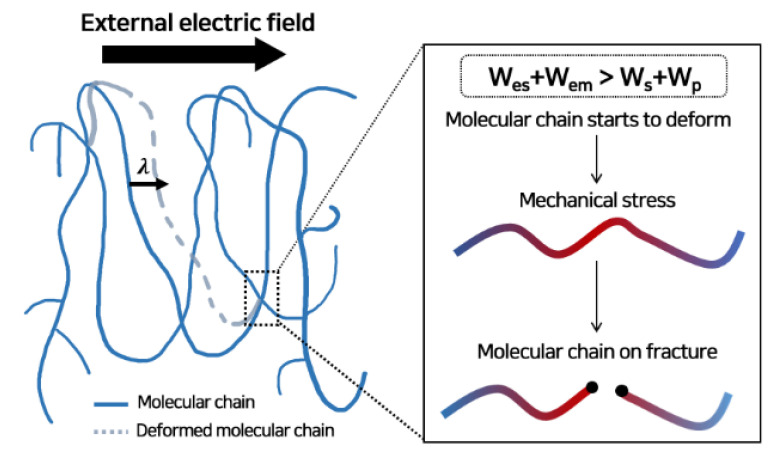
Schematic representation of MCD model with threshold energy condition. After the electrical energy exceeds the mechanical threshold energy, the molecular chain begins to deform, resulting in an electro-fracture at the electrical stress concentration point (red-colored area). Breakdown starts from this point where the physical crack has occurred.

We numerically calculated the breakdown strength based on the space charge and electric field distribution with time. In the previous study, the MCD model was used to calculate the breakdown strength [37]. This conventional MCD model explains that the molecular chain of polymeric insulators is deformed by the electric force acting on the trapped charge. This model is suitable for a continuously increasing voltage with a constant ramp rate. However, this conventional model predicts an excessively smaller value than the actual breakdown strength when the CV is applied, as depicted in Figure S10. To overcome the limits of this conventional model expressed as Equation (S6), an additional threshold energy condition was proposed, based on the electro-mechanical energy relation as [38]:

*W_es_* + *W_em_* > *W_s_* + *W_p_* (7)

where *W_es_* = 1/2*ε*_0_*ε_r_E*^2^ is the stored electric field energy per unit volume, *W_em_* = *ε*_0_*ε_r_E*^4^/8*Y* is the mechanical stress induced by the electric field with the yield strength, *Y*, *W_s_* is the surface energy that must be overcome to develop a crack, proportional to the fracture toughness, *G* = 6500 J/m^2^, and *W_p_* is the mechanical energy dissipated by the crack, proportional to the yield strength. *W_es_*, *W_em_*, and *W_p_* are the energies proportional to the volume of the crack, whereas *W_s_* is the energy proportional to the surface of the crack. When the electrical energy exceeds the mechanical threshold energy released by the crack, the molecular chain starts to deform by the electric force, as depicted in Figure 7. The yield strength used in this model was obtained by interpolating the temperature-varying values in the experimental study (further details can be found in Supplementary Material, S2). With an additional threshold energy condition, we proposed the modified molecular chain displacement (M-MCD) model as:

(8) $$\frac{d\lambda (x,t)}{dt}=\{\begin{array}{c} \mu_{mol}E(x,t)-\frac{\lambda (x,t)}{\tau_{mol}},\;\;\;\;\;if:\;W_{s}+W_{em}>W_{s}+W_{p}\\ 0,\;\;\;\;\;\;\;\;\;\;\;\;\;\;\;\;\;\;\;\;\;\;\;\;\;\;\;\;\;\;\;\;\;\;\;if:\;W_{s}+W_{em}\leq W_{s}+W_{p} \end{array}$$

where *λ* is the displacement of the molecular chain in nm, *μ_mol_* is the mobility of the molecular chain in m^2^/V∙s, and *τ_mol_* is the relaxation time in s. For LDPE, the critical displacement length for breakdown initiation is 23 nm [1,25].

### 4.2. Analysis Results of Breakdown Strength

**Figure 8:**
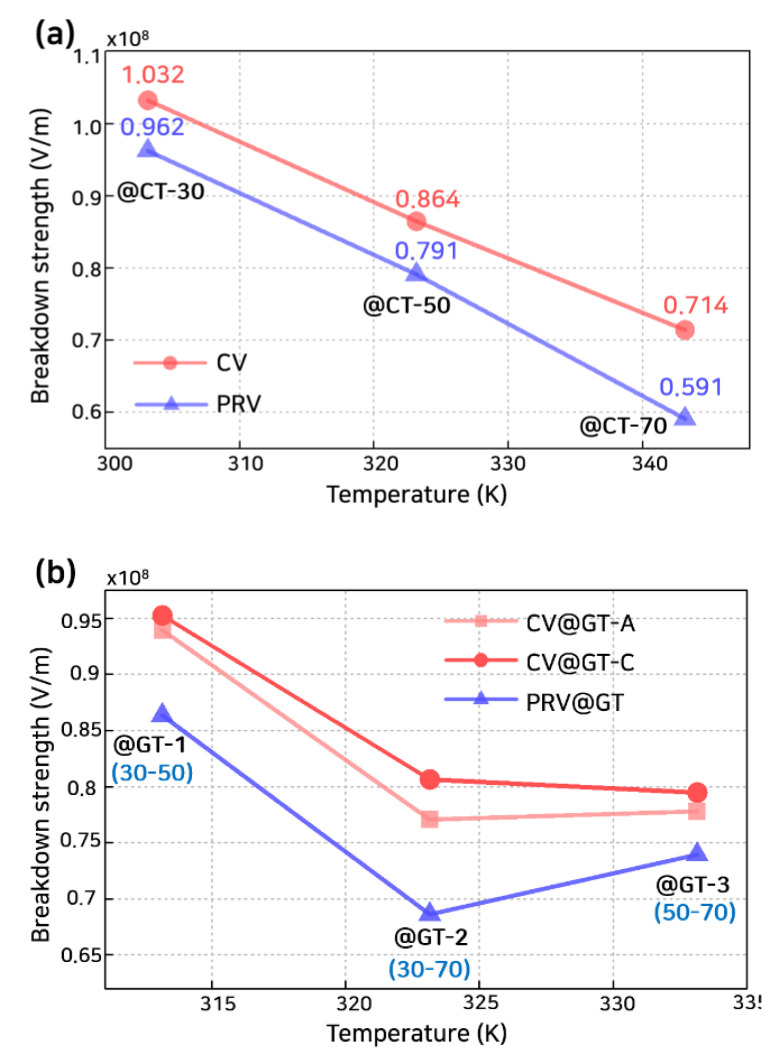
Breakdown strength of (**a**) @CT-30, 50, and 70 and (**b**) @GT-1, 2, and 3. CT and GT denote a constant temperature and temperature gradient that exist inside the polymeric insulator, respectively.

**Figure 9:**
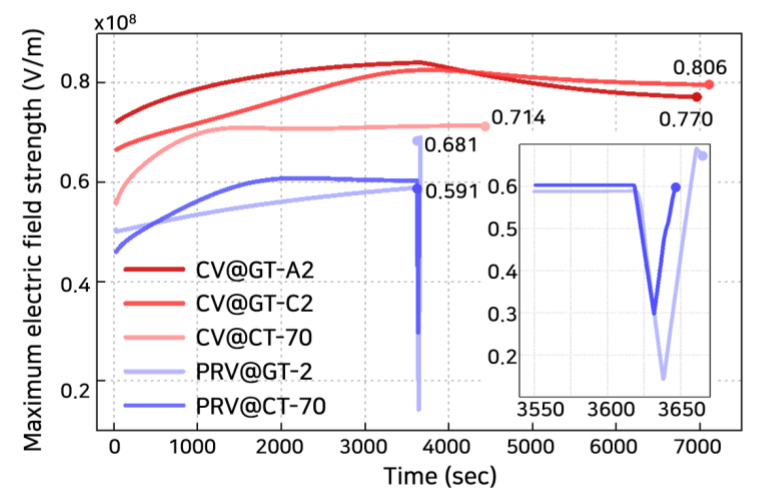
Maximum electric field strength inside the polymeric insulator varies with time before breakdown initiation: CV@GT-A2, CV@GT-C2, CV@CT-70, PRV@GT-2, and PRV@CT-70.

The breakdown strength was calculated by using the M-MCD model as depicted in Figure 8 and Figure 9. These numerical analysis results were verified through the experiments in the previous study [39,40,41] (detailed results to verify the numerical analysis model are included in the Supplementary Material, S2-3). Figure 8a shows the calculated breakdown strength under a constant voltage with constant temperature, CV@CT, and a polarity-reversal voltage with constant temperature, PRV@CT. The breakdown strength decreased as temperature increased with a PRV. The temperature was significant for reducing the breakdown strength of polymeric insulators, irrespective of the applied voltage waveform. Compared to CV, with PRV, the breakdown strength was decreased as described in recent studies [11,17,41,42,43]. The critical environment for LDPE was when the temperature in the entire polymeric insulator reached 70 °C with PRV (PRV@CT-70). Here, the breakdown strength decreased by about 82% from 71.4 kV/mm (CV@CT-70) to 59.1 kV/mm (PRV@CT-70). The breakdown strength decreased by about 93% for 30 °C and 91% for 50 °C. Similarly, for the case of polymeric insulator pre-stressed by the polarity reversal voltage, breakdown strength decreased 81%~95% (detailed results for breakdown strength of pre-stressed polymeric insulator are included in the Supplementary Material, Figure S9) [41].

Figure 8b shows that the calculated breakdown strength under a constant voltage with a temperature gradient, CV@GT, and a polarity reversal voltage with temperature gradient, PRV@GT. Temperature gradient cases can be divided into heated anode cases (@GT-A1, -A2, and -A3) and heated cathode cases (@GT-C1, -C2, and -C3). Numbers 1 to 3 indicate the cases at 30 °C~50 °C; 30 °C~70 °C; and 50 °C~70 °C, respectively. The breakdown strength decreased as the temperature gradient increased [44]. The breakdown strength was slightly larger for CV@GT-Cs rather than CV@GT-As in Figure 8b. The difference between the heated cathode and anode cases has been reported in previous studies [44,45]. The difference between @GT-Cs and @GT-As originated from the position of the maximum electric field, as depicted in Figure 3 and Figure 4. For @GT-As, the maximum electric field was located closer to the low-temperature region. Although the temperature gradient was expected to reduce the breakdown strength significantly, it had an approximate intermediate value between the two cases related to the ends’ temperature of the polymeric insulator. The breakdown strength was 77.0 kV/mm (CV@GT-A2) and 80.6 kV/mm (CV@GT-C2) between 100.3 kV/mm (CV@CT-30) and 71.4 kV/mm (CV@CT-70), respectively. With a temperature gradient, the maximum electric field occurred near the low-temperature region. Hence, the mechanical threshold energy was higher than when a high temperature was applied to the entire polymeric insulator. For PRV@GT-2, the breakdown strength decreased rapidly to 68.1 kV/mm from CV@GT-A2 and CV@GT-C2 cases. Herein, PRV had a more significant effect on reducing the breakdown strength of the @CT cases than @GT cases. On average, the decrement rates in breakdown strength due to the polarity-voltage effects were 8.0% and 10.8% when a temperature gradient exists and when the temperature was constant, respectively.

Figure 9 shows the change in the maximum electric field strength inside the polymeric insulator with time before breakdown initiation. The breakdown occurred at a reduced electric field strength when the PRV was applied compared to when CV was applied. Under PRV, the breakdown occurred slightly after the voltage polarity changed. When CV was applied, the maximum electric field inside the polymeric insulator continuously increased and then progressively decreased until the equilibrium state was attained. In previous studies, the time to reach the breakdown is longer in the cathode-heated case than in the anode-heated case [45]. After the voltage was applied, for CV@CT-2 and CV@AT-2, the breakdown was reached after 7120 and 7000 s, respectively.

**Figure 10:**
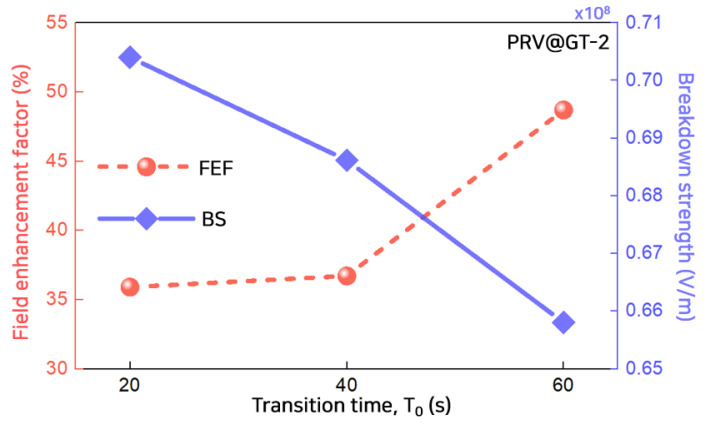
Changes in field enhancement factor and breakdown strength with transition time *T*_0_, 20 s~60 s under PRV@GT-2. FEF denotes a field enhancement factor and BS denotes a breakdown strength.

Figure 10 shows the changes in the electric field enhancement factor and breakdown strength with the transition time, *T*_0_: 20 s~60 s under PRV@GT-2. The field enhancement factor (*FEF*) represents the distortion of the local electric field due to the accumulated space charge. *FEF* can be calculated using the maximum electric field and the applied electric field described as [14]:

(9) $$FEF=\frac{E_{max}-E_{appl}}{E_{appl}}\times 100\%$$

where *E_max_* is the maximum electric field and *E_app_* is the applied electric field. As *T*_0_ increased, the EFE inside the polymeric insulator increased. As the polarity reversal occurred slowly, the remaining homo-charge decreased and the injected hetero-charge increased, causing severe electric field enhancement near the electrode. The breakdown strength, however, decreased with *T*_0_. The decrease in breakdown strength can be explained as the accumulated hetero-charge intensifies the local electric field distortion as *T*_0_ increases. Moreover, these results agree well that the breakdown strength increases with the voltage ramp rate analyzed in our previous research results [40].

## 5. Discussion

**Figure 11:**
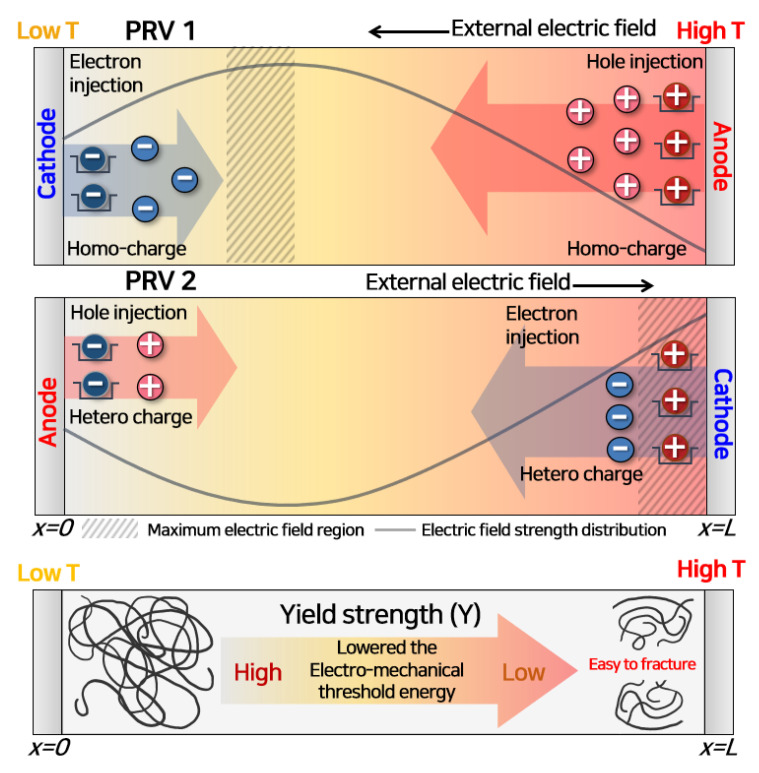
Schematic representation of the breakdown mechanism under a PRV with a temperature gradient (PRV@GT-2).

Based on the space charge and electro-mechanical energy distribution calculated using the numerical analysis model, it was possible to explain why the breakdown strength weakened when the polarity-reversal voltage was applied with a temperature gradient, as depicted in Figure 11.

In PRV 1, when the positive voltage was applied, many holes were injected from the anode (high-temperature region). Most of the holes were trapped adjacent to the electrode so that the maximum electric field strength appeared in the low-temperature region. When a large amount of space charge accumulates inside the molecular chain, the inter-atomic distance increases. Additionally, the enhancement of the local electric field easily destroys the molecular chain due to partial damage [41,46].

In PRV 2, the charge having an opposite polarity to the trapped charge was injected. Then, the maximum electric field appeared near the anode due to the interaction between hetero-charges [36]. At this time, the imbalance in the electrical and mechanical energies accelerated breakdown inside the polymeric insulator. The mechanical threshold energy required to initiate MCD is proportional to the yield strength. The yield strength decreases as the temperature increases [47,48,49]. Therefore, low mechanical threshold energy and the maximum electric field appeared simultaneously at the anode (high-temperature region). As a result, the magnitudes of electrical and mechanical energies were reversed in PRV 2, as depicted in Figure S6.

The slope of polarity reversal voltage and the temperature gradient strongly contribute to lowering the breakdown strength with the thermal effect. The yield strength of the polymeric insulator decreases as the electro-mechanical threshold energy is lowered. As the mechanical and electrical stresses are concentrated in the region where the molecular chain has low threshold energy, therefore, one can build an insulation design scheme using this proposed analysis.

## 6. Conclusions

In this study, the space charge behavior and the breakdown strength were numerically analyzed under various conditions in which a temperature gradient exists and the polarity of the applied voltage changes. We analyzed the space charge behavior with the thermal effect inside the polymeric insulator by fully coupled the BCT and the heat transfer models. Moreover, we proposed the M-MCD model, considering the electro-mechanical threshold energy to predict the breakdown strength. This model was successfully tested under various severe environments. Additionally, the numerical results with the M-MCD model were verified by comparing those with experiments in previous research.

Unlike the conventional calculating method for breakdown strength, our newly proposed M-MCD model can effectively calculate the breakdown strength considering the space charge behavior, including the temperature and applied voltage effects. In particular, we can successfully predict the breakdown strength with the constant voltage while the conventional approach fails.

Moreover, we revealed the mechanism that the breakdown strength quantitatively weakened while the PRV was applied with a temperature gradient by employing the imbalances of electrical and mechanical energies. This breakdown mechanism was discovered using the M-MCD model, which was a combination of the conventional MCD model and the proposed threshold energy condition. This proposed methodology can predict the breakdown strength of polymeric insulators in various environments, and suggest enhancing the electrical characteristics for developing new polymeric materials.

## Figures and Tables

**Table 1:** Various conditions analyzed in this numerical simulation.

Applied Voltage	Temperature (°C)	
	Constant	Gradient (Anode-Cathode)
One-directional constant voltage (CV)	30 (@CT30) 50 (@CT50) 70 (@CT70)	Heated Anode 30~50 (@GT-A1) 30~70 (@GT-A2) 50~70 (@GT-A3) Heated Cathode 50~30 (@GT-C1) 70~30 (@GT-C2) 70~50 (@GT-C3)
Polarity-reversal voltage (PRV)	30 (@CT30) 50 (@CT50) 70 (@CT70)	30~50 (@GT-1) 30~70 (@GT-2) 50~70 (@GT-3)

## Data Availability

The data presented in this study are available in Supplementary Material. The additional data presented in this study are available on request from the corresponding author.

## Supplementary Materials

The following are available online at https://www.mdpi.com/article/10.3390/polym13162746/s1, and supplementary data file includes the detailed parameters and results of numerical simulation. (S1. Model and selection of parameters for BCT model with temperature effect, S2. Breakdown strength prediction).

## Abbreviations

CT	Constant temperature
CV	Constant voltage
FEF	Field enhancement factor
MCD	Molecular chain displacement
M-MCD	Modified molecular chain displacement
PRV	Polarity-reversal voltage
