# Supplementary material for: Modified Molecular Chain Displacement Analysis Employing Electro-Mechanical Threshold Energy Condition for Direct Current Breakdown of Low-Density Polyethylene
Source: Polymers (Basel). 2021 Aug 16;13(16):2746. doi: 10.3390/polym13162746 (PMC8401579; doi:10.3390/polym13162746)
Supplement: Supplementary file 1 [file polymers-13-02746-s001.zip › polymers-1313404-supplementary.pdf]

Supplementary Material for:

Modified Molecular Chain Displacement Analysis Employing Electro-Mechanical Threshold Energy Condition for Direct Current Breakdown of Low-density Polyethylene

Minhee Kim and Se-Hee Lee
School of Electronic and Electrical Engineering, Kyungpook National University, Daegu, 41566, Korea

*Correspondence: shlees@knu.ac.kr

S1. Model and selection of parameters for bipolar charge transport (BCT) model with temperature effect

S1-1: Numerical simulation conditions

This numerical simulation analyzed various environments related to temperature and applied voltage waveforms. The details about these various environments are as follows:

1. **Temperature distribution inside**
   A. The temperature of the polymeric insulator is constant: 30℃, 50℃, and 70℃
   B. A temperature gradient exists inside the polymeric insulator: Δ20℃ and Δ40℃ (30℃~50℃, 30℃~70℃, 50℃~70℃). These cases can be divided into the heated anode and cathode, respectively.

2. **Applied voltage**
   A. One-directional constant voltage : CV (Figure S1a)
   B. Polarity reversal voltage : PRV (Figure S1b)

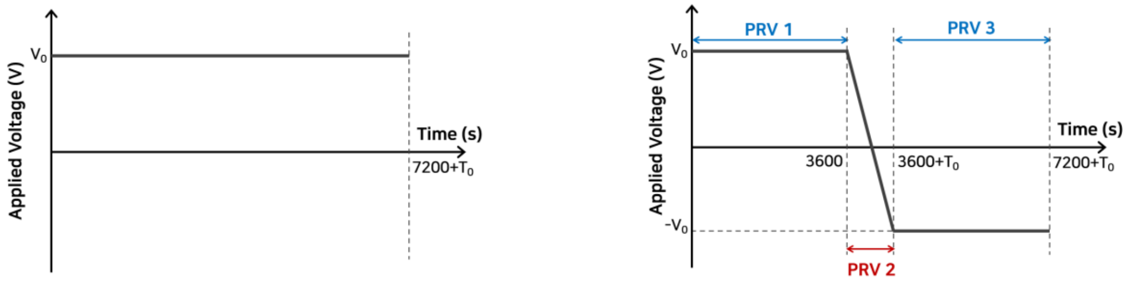

(a) (b)

Figure S1. Two kinds of applied voltage waveform: (a) one-directional constant (CV) and (b) polarity-reversal voltages (PRV).

S1-2: Detailed charge transport process

The trapping probability, P_T, is the probability that the charges are trapped in deep traps and represents how often the charges are captured in one second as

$$P_T = \frac{\mu_0 e N_T}{\varepsilon_0 \varepsilon_r} \quad \text{(S1)}$$

which is proportional to the mobility of the charge carriers and the distribution density of the traps, N_T. Further, it is inversely proportional to the relative permittivity of the insulator ε_r. The probability of detrapping affects the trapped charge drifting back into the conduction band as

$$P_{DT} = v_{ATE} \exp(-\frac{E_T}{k_B T}) \quad \text{(S2)}$$

where v_{ATE} is the escape frequency expressed as k_BT/h and E_T is deep trap energy. After the detrapping process, the charges move in the direction of the electric field or vice versa to cause a drift current. The recombination probability of free mobile charges is proportional to the sum of the mobilities of the electrons and holes. The Langevin-type recombination can express this electron-hole recombination process, and the recombination coefficients are a function of the mobilities of the charge carriers based on the Shockley-Read-Hall recombination model as

$$R_{\mu e \mu h} = \frac{q_{free(e)} q_{free(h)} (\mu_{0(e)} + \mu_{0(h)})}{\varepsilon_0 \varepsilon_r}$$
$$R_{\mu e t h} = \frac{q_{free(e)} q_{free(h)} \mu_{0(e)}}{\varepsilon_0 \varepsilon_r} \quad \text{(S3)}$$
$$R_{t e \mu h} = \frac{q_{free(e)} q_{free(h)} \mu_{0(h)}}{\varepsilon_0 \varepsilon_r}$$

where the subscript (μ) and (t) denote the mobile charges and trapped charges, respectively. $R_{\mu e\mu h}$ is the recombination probability of free electrons and free holes. $R_{\mu eth}$ and $R_{te\mu h}$ represent the probabilities of the combination of free charges and trapped charges. These values are proportional to the mobility, density of trapped charge q_{trap}, and density of free charge q_{free}. The reaction terms can be expressed as

$$
\begin{aligned}
S_{\mu e} &= -P_{T(e)} q_{free(e)} \left(\frac{1-q_{trap(e)}}{eN_{T(e)}}\right) + P_{DT(e)} q_{trap(e)} - R_{\mu e\mu h} q_{free(e)} q_{free(h)} - R_{\mu eth} q_{free(e)} q_{trap(h)} \\
S_{te} &= P_{T(e)} q_{free(e)} \left(\frac{1-q_{trap(e)}}{eN_{T(e)}}\right) - P_{DT(e)} q_{trap(e)} - R_{te\mu h} q_{free(e)} q_{free(h)} \\
S_{\mu h} &= -P_{T(h)} q_{free(h)} \left(\frac{1-q_{trap(h)}}{eN_{T(h)}}\right) + P_{DT(h)} q_{trap(h)} - R_{\mu e\mu h} q_{free(h)} q_{free(e)} - R_{te\mu h} q_{free(h)} q_{trap(e)} \\
S_{th} &= P_{T(h)} q_{free(h)} \left(\frac{1-q_{trap(h)}}{eN_{T(h)}}\right) - P_{DT(h)} q_{trap(h)} - R_{\mu eth} q_{free(h)} q_{free(e)}
\end{aligned}
\qquad \text{(S4)}
$$

where $S_{\mu e}$, S_{te}, $S_{\mu h,}$ and S_{th} represent the reaction terms for the mobile electrons, trapped electrons, mobile holes, and trapped holes, respectively.

S1-3: Detailed coefficients for BCT model

The space charge transport process is strongly affected by the temperature inside the polymeric insulator. The temperature changes the charge carrier mobility and deep trap energy and density. The mobilities of electrons and holes are the functions of temperature and electric field strength. These mobilities increase with temperature (eqs S5a and S5b). All values used in this numerical simulation were derived from the experimental results of the previous studies [1,2].

- Mobility of electrons

$$\mu_e(T) = 3.77\times10^{-3}\exp(-7529/T) \qquad \text{(S5a)}$$

- Mobility of holes

$$\mu_h(T) = \mu_0(\mathrm{E})6.11\times10^{-4}\exp(-7751/T) \qquad \text{(S5a)}$$

where $\mu_e(T)$ and $\mu_h(T)$ are the electron and hole mobilities in $m^2/V\cdot s$, respectively. μ_0(E) is the empirical value that depends on the electric field strength included in the holes' mobility (Figure S2)[2]. This value nonlinearly decreases with electric field strength.

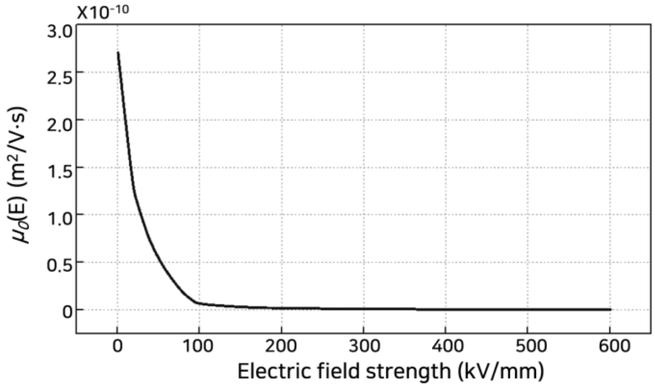

Figure S2. μ_0**(E) is a function of the electric field strength.**

Table S1. Deep trap energy and density

Quantity	Temperature (°C)		
	30	50	70
Deep trap energy (eV)	0.987	1.019	1.072
Deep trap density × 10^{20} ($1/eV\cdot m^3$)	7.236	5.436	4.934

The deep trap energy and density were obtained from the experimental paper (Table S1) [1]. As the temperature increases, the deep trap energy increases, and the deep trap density decreases. Other parameters employed for the BCT model are summarized in Table S2.

Table S2. Parameters used in the BCT model

Parameter	Unit	Value
Relative permittivity	-	2.3
Thickness	μm	200
Injection barrier height (Anode)	eV	1.27
Injection barrier height (Cathode)	eV	1.31

S1-4: Detailed coefficients for Heat Transfer Equation

Figure S3 shows the variation of the thermal conductivity, *k* in W/m·K as a function of temperature from the experimental study [3].

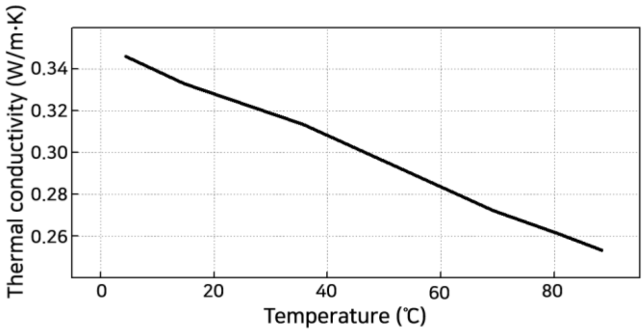

Figure S3. Thermal conductivity as a function of temperature.

S1-5: Numerical analysis results of space charge behavior under Temperature gradient (△40°C)

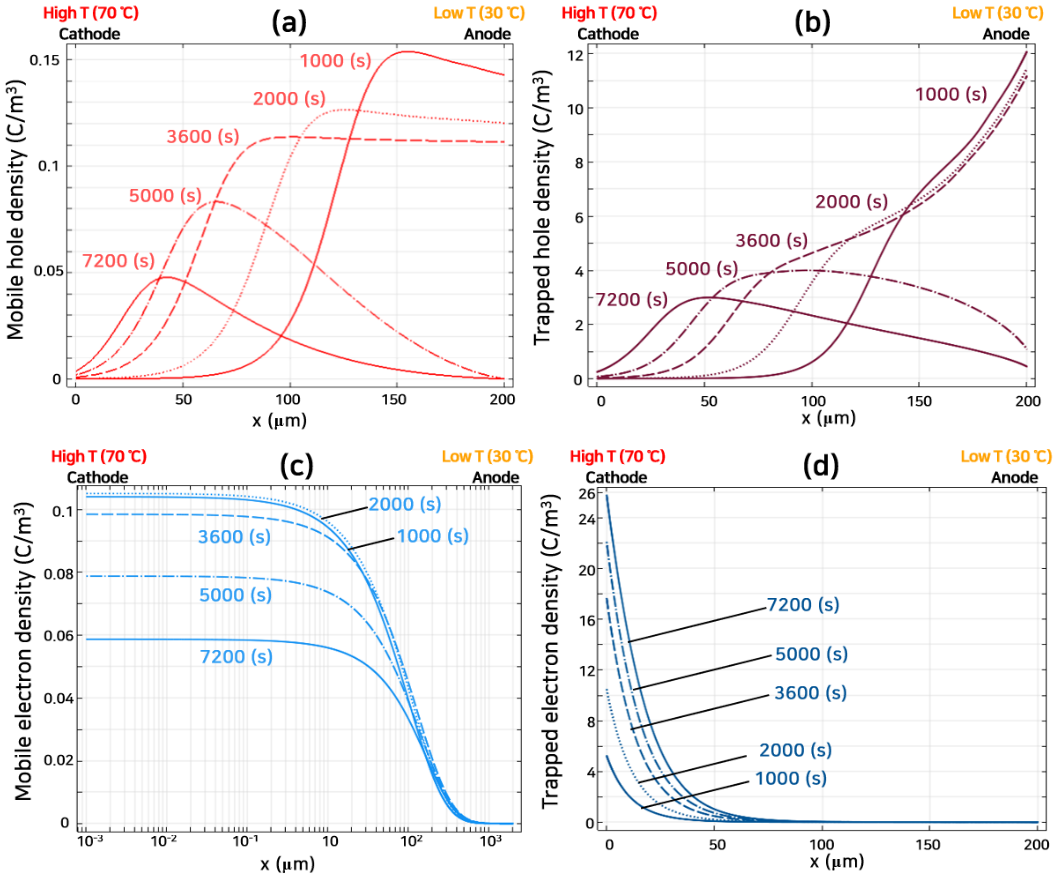

Figure S4. Space charge distribution with time (a) mobile hole, (b) trapped hole, (c) mobile electron, and (d) trapped electron in CV@GT-A2 and V_0=10 kV.

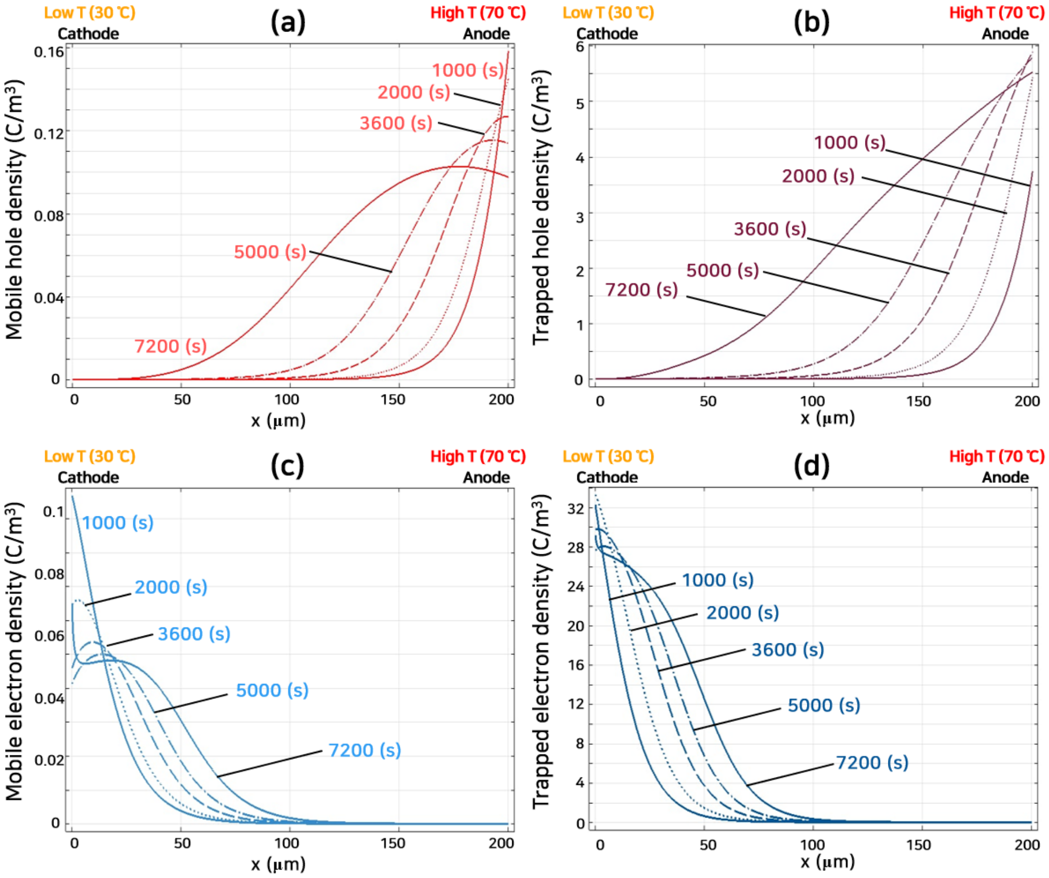

Figure S5. Space charge distribution with time (a) mobile hole, (b) trapped hole, (c) mobile electron, and (d) trapped electron in CV@GT-C2 and V_0=10 kV.

S2. Breakdown strength prediction

S2-1: Modified molecular chain displacement model

To predict the breakdown strength, we employed the threshold energy condition. We already calculated the breakdown strength under continuously increasing voltage using numerical simulation in the previous study [4,5]. Equation 8 in the manuscript includes all the conditions about eq S6 and eq S7 as

$$\frac{d\lambda(x,t)}{dt} = \mu_{mol} E(x,t) - \frac{\lambda(x,t)}{\tau_{mol}} \quad \text{(S6)}$$

$$W_{es} + W_{em} > W_s + W_p \quad \text{(S7)}$$

The molecular chain displacement continuously increases only if the electrical energy exceeds the mechanical threshold energy. μ_{mol} and τ_{mol} used in this equation depend on the temperature, as shown in Table S3. W_{es}, W_{em}, and W_p are the energies proportional to the volume of the crack, whereas W_s is the energy proportional to the surface area of the crack. The volume and surface area of the crack is proportional to λ and the length of the molecular chain, 0.84 μm. The yield strength used in this model was obtained by interpolating the temperature-varying values in the experimental study: 23.6 MPa at 23°C, 16.0 MPa at 40°C, and 9.5 MPa at 70°C [6].

Table S3. Parameters used in the breakdown strength prediction model

Quantity	Temperature (°C)		
	30	50	70
Mobility of molecular chain, μ_{mol} $\times 10^{-17}$ (m^2/V·s)	0.2275	2.4676	12.023
Relaxation time, τ_{mol} (s)	5818.8	4623.1	683.9

S2-2: Energy distribution calculation

Figure S6 shows that the calculation results of energy distribution inside the LDPE of PRV@GT-2. W_{mech} indicates the sum of W_s and W_p as the mechanical threshold energy. It is proportional to yield strength, *Y*, that changes with temperature. W_{mech} had a large value at $x = 0\ \mu m$ with a low temperature and a small value at $x = 200\ \mu m$ with a high temperature. The temperature distribution inside the LDPE changed for the first few seconds after the PRV was applied. After that, the equilibrium state for temperature was maintained, so the distribution of W_{mech} hardly changed before breakdown initiation. W_{elec} indicates the sum of W_{es} and W_{em} as electrical energy. At 3,650 s, the maximum value of W_{elec} occurred at x = 200 μm, and had a smaller value than W_{mech}. At 3,655 s, W_{elec} exceeded the mechanical threshold energy, W_{mech} at x = 200 μm. Therefore, λ started to increase and led to breakdown as described in the eqs S2 and S3.

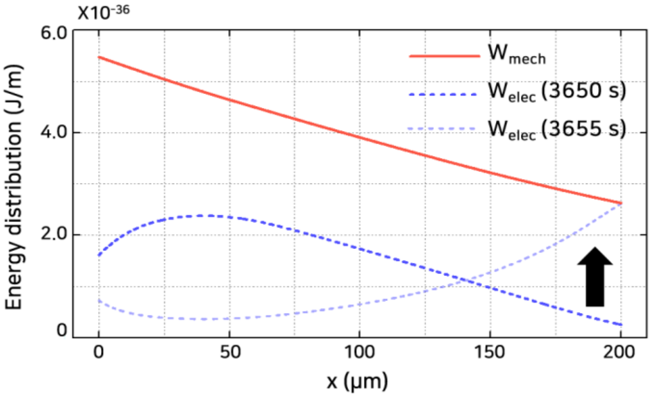

Figure S6. The calculation results of energy inside the LDPE of PRV@GT-2, W_{mech} means the sum of W_s and W_p, and W_{elec} means the sum of W_{es} and W_{em}.

S2-3: Breakdown strength calculation: validity of the numerical model

In Figures S7 and S8, the calculated breakdown strengths are depicted employing the M-MCD model under continuously increasing voltage. These calculated results agreed with those experimentally determined in a previous studies [7–9]. The breakdown strength increased with the ramp rate but decreased with temperature and thickness following the power law. In Figure S8, the circle, triangles, and squares indicate the breakdown strengths at a thickness of 100 μm, 1,000 V/s, 25 μm at 500 V/s, and 150 μm at 2,000 V/s respectively.

In Figure S9, the calculated breakdown strengths are depicted employing the M-MCD model for pre-stressed samples with thickness 80 μm. The pre-stressed electric field varies from 0 to 50 kV/mm. After 20 minutes of the pre-stress process, a voltage of opposite polarity was applied at 500V/s. The breakdown strength calculated through numerical analysis is slightly larger than the experimental result. The overall trend is in good agreement with experimental results [10].

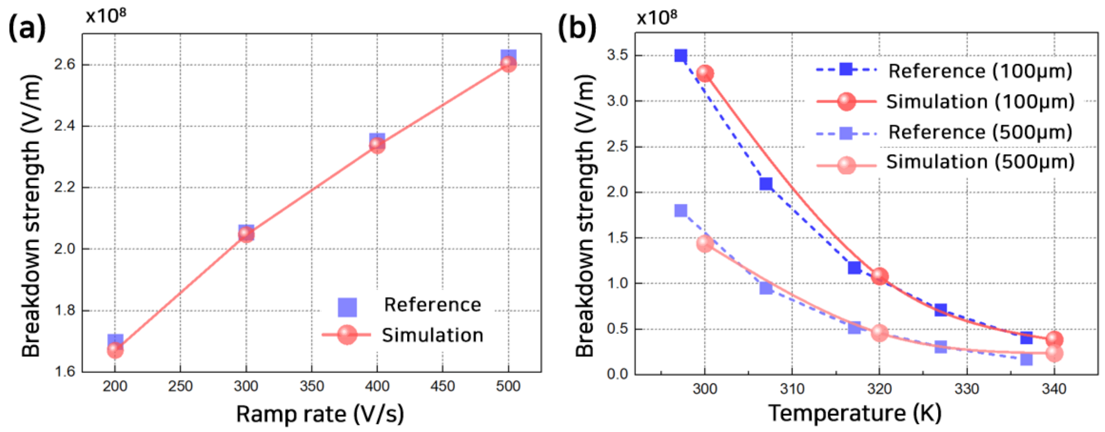

Figure S7. Comparison between numerical and experimental results[7] of breakdown strength under continuously increasing voltage in the previous literature. Breakdown strength with (a) ramp rate at 30°C. (b) varying temperature and thickness (100, 500 μm)

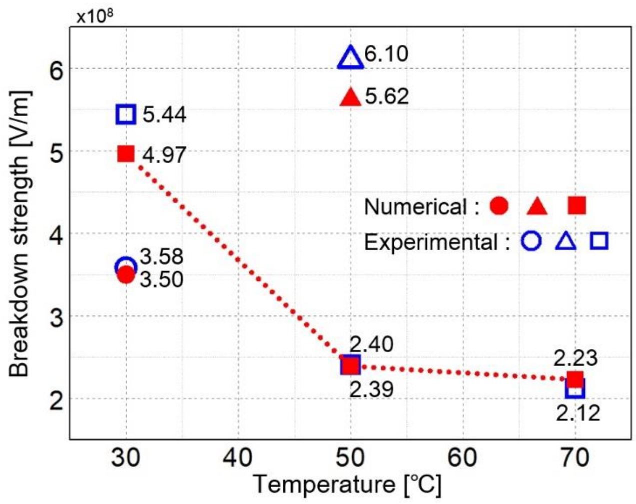

Figure S8. Comparison between numerical and experimental results of breakdown strength under continuously increasing voltage in the previous literature and experimental results obtained from previous researches [5,7–9].

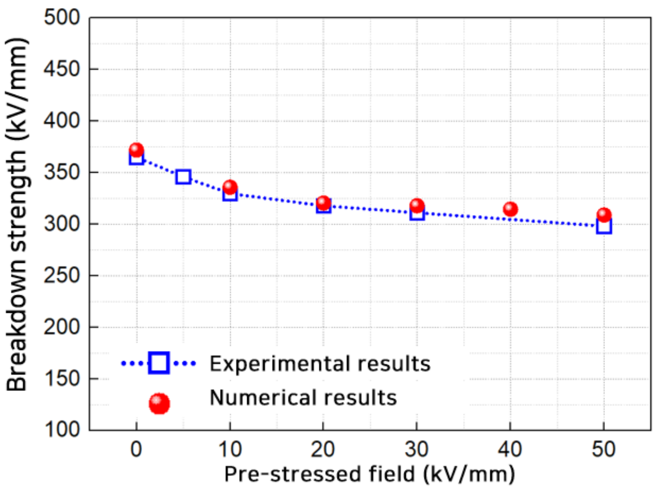

Figure S9. Comparison between numerical and experimental results of breakdown strength under polarity reversal pre-stressed electric field in the previous literature [10].

S2-4: Breakdown strength calculation: comparison with other models

In Figure S10, the breakdown strengths are depicted to compare the M-MCD model with the conventional MCD model under CV. As mentioned in the manuscript, the conventional MCD model excluded the threshold energy condition described in eq S3. This threshold energy condition plays a meaningful role in calculating the breakdown strength, especially under CV. With the conventional MCD model, the breakdown always occurs after a sufficient time regardless of the applied voltage magnitude, V_0. Therefore, the breakdown strength with the conventional MCD model was much smaller than that with the M-MCD model under both @CT and @GT.

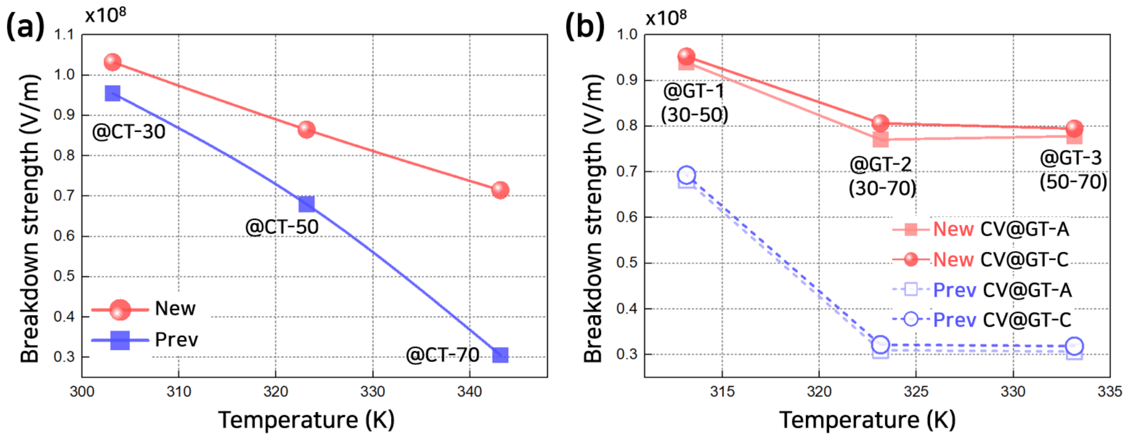

Figure S10. Comparison between calculated breakdown strength employing new threshold energy condition, M-MCD model, and conventional MCD model. "New" describes that the calculated results derived from the M-MCD model, and "Prev" describes that the results derived from the conventional MCD model. (a) shows the breakdown strength of CV@CTs and (b) shows that of CT@GTs.

Reference

1. Li, Z.L.; Du, B.X.; Yang, Z.R.; Han, C.L. Temperature dependent trap level characteristics of graphene/LDPE nanocomposites. *IEEE Trans. Dielectr. Electr. Insul.* **2018**, *25*, 137–144.
2. Zhou, C. Space Charge Dynamics in Polyethylene under Periodical High Voltage Electric Fields, University of Southampton, 2017, Vol. 142.
3. Eiermann, K.; Hellwege, K. -xH. Thermal conductivity of high polymers from −180°C. to 90°C. *J. Polym. Sci.* **1962**, *57*, 99–106.
4. Kim, M.; Kim, S.-H.; Lee, S.-H. Finite Element Analysis of the Breakdown Prediction for LDPE Stressed by Various Ramp Rates of DC Voltage Based on Molecular Displacement Model. *Energies* **2020**, *13*, 1320.
5. Kim, M.; Kim, S.H.; Lee, S.H. Numerical Prediction of DC Breakdown Characteristics in LDPE with Current Profile as Critical Index. *IEEE Access* **2020**, *8*, 200051–200062.
6. Merah, N.; Saghir, F.; Khan, Z.; Bazoune, A. Effect of temperature on tensile properties of HDPE pipe material. *Plast. Rubber Compos.* **2006**, *35*, 226–230.
7. Min, D.; Li, S.; Ohki, Y. Numerical simulation on molecular displacement and DC breakdown of LDPE. *IEEE Trans. Dielectr. Electr. Insul.* **2016**, *23*, 507–516.
8. Song, S.; Zhao, H.; Yao, Z.; Yan, Z.; Yang, J.; Wang, X.; Zhao, X. Enhanced electrical properties of polyethylene-graft-polystyrene/LDPE composites. *Polymers (Basel).* **2020**, *12*.
9. Wu, K.; Chen, X.; Liu, X.; Wang, X.; Cheng, Y.; Dissado, L.A. Study of the space charge behavior in polyethylene nano-composites under temperature gradient. In Proceedings of the Proceedings of the International Symposium on Electrical Insulating Materials; Institute of Electrical Engineers of Japan, 2011; pp. 84–87.
10. Han, C.; Du, B.X.; Li, J.; Li, Z.; Tanaka, T. Investigation of charge transport and breakdown properties in XLPE/GO nanocomposites part 2: Effect of polarity reversal. *IEEE Trans. Dielectr. Electr. Insul.* **2020**, *27*, 1213–1221.
